# Static Balance and Chair-Rise Performance in Neurogeriatric Patients: Promising Short Physical Performance Battery-Derived Predictors of Fear of Falling

**DOI:** 10.3389/fmed.2022.904364

**Published:** 2022-06-21

**Authors:** Katharina Scholz, Johanna Geritz, Jennifer Kudelka, Marten Rogalski, Katharina Niemann, Corina Maetzler, Julius Welzel, Michael Drey, Tino Prell, Walter Maetzler

**Affiliations:** ^1^Department of Neurology, University Hospital Schleswig-Holstein, Kiel, Germany; ^2^Department of Medicine IV, Geriatrics, University Hospital of LMU Munich, Munich, Germany; ^3^Department of Geriatrics, Halle University Hospital, Halle, Germany

**Keywords:** fear of falling, mobility, static balance, sit-to-stand, geriatrics, BIA, depressive symptoms

## Abstract

**Background:**

Fear of falling (FOF) negatively affects health-related quality of life and is common in neurogeriatric patients, however, related parameters are not well understood. This study investigated the relationship between FOF, physical performance (as assessed with the Short Physical Performance Battery and its subscores) and other aspects of sarcopenia in a sample of hospitalized neurogeriatric patients.

**Methods:**

In 124 neurogeriatric patients, FOF was assessed with the Falls Efficacy Scale International (FES-I). Physical performance was measured using the Short Physical Performance Battery (SPPB) including walking duration, balance and five times sit-to-stand task (5xSST) subscores. Appendicular skeletal muscle mass (ASMM) was estimated with the cross-validated Sergi equation using Bioelectrical impedance analysis measures. The Depression im Alter-Skala (DIA-S) was used to assess depressive symptoms. Multiple regression models with FES-I score as outcome variable were computed using backward selection with AICc as selection criterion, including: (i) SPPB total score, ASMM/height^2^, grip strength, age, gender, positive fall history, number of medications, use of a walking aid, DIA-S score and Montreal Cognitive Assessment (MoCA) score; and (ii) SPPB subscores, ASMM/height^2^, grip strength, age, gender, positive fall history, number of medications, DIA-S score and MoCA score, once with and once without including use of a walking aid as independent variable.

**Results:**

Lower SPPB total score, as well as lower SPPB balance and 5xSST subscores were associated with higher FES-I scores, but SPPB walking duration subscore was not. Moreover, DIA-S, number of medications and use of a walking aid were significantly associated with FOF.

**Conclusion:**

Our preliminary results suggest that -if confirmed by subsequent studies- it may be worthwhile to screen patients with low SPPB balance and 5xSST subscores for FOF, and to treat especially these mobility deficits in neurogeriatric patients with FOF. Moreover, training neurogeriatric patients to use their walking aids correctly, critical evaluation of medication and treating depressive symptoms may further help reduce FOF in this highly vulnerable cohort.

## Introduction

Fear of falling (FOF) is a common and serious condition affecting 21−85% of older adults (1). FOF is also prevalent in individuals without a fall history and can predict future falls (2, 3). FOF could evoke reasonable caution in individuals with a high fall risk, but may also be a debilitating symptom that could cause disproportionate activity avoidance (2, 4–8). This may lead to a deconditioning effect on muscle strength and balance, aggravating a downward spiral towards functional decline (2, 5, 9, 10). Neurological diseases such as stroke/small vessel disease, vertigo, Parkinson's disease (PD) and polyneuropathy (PNP) have been identified as risk factors for falls (2, 11–13) with the prevalence of falls among neurological in-patients being almost twice as high as in an age-matched population (14). FOF was identified as a risk factor for future falls in neurological patients (14, 15) and is negatively correlated with health-related quality of life in patients with PD (16, 17) and stroke (18). Previous studies in community-dwelling older adults have shown that low scores in the Short Physical Performance Battery (SPPB), a composite test measuring domains of physical performance like walking (with walking duration), static balance (in standing positions) and transfer (five times sit-to-stand-to-sit task (5xSST)) (19–21), are associated with higher FOF (22–24) and may even be (in combination with self-rated disability) predictive for the development of FOF (25). Additionally, a study in patients with stroke found an association between the SPPB and fall-related self-efficacy (26) (the confidence of a person not to fall), which is related to – but not identical with FOF (27). The SPPB is a useful tool in geriatric assessments as it is predictive for falls, all-cause mortality, institutionalization and disability (19, 28–30). However, many studies on FOF in neurological patients do not include the SPPB as a physical performance measure (17, 18, 31–35), even though its use may have several advantages. Balance and lower limb strength (which is needed to successfully rise from a chair and keeping a stand-up position (36)) have been found to be associated with FOF in patients with stroke (18, 35) and PD (32, 34). Further, self-reported difficulty in rising from a chair has been previously linked to higher FOF in patients with PD (17). Adding to the importance of balance with regard to FOF, an intervention study was able to show that balance training reduces FOF in older institutionalized patients (37). Evidence on the relationship between FOF and gait speed (which is represented by walking duration in the SPPB) differs in the literature (32, 38–41). For example, a study in patients with PD found a correlation between FOF and gait speed in bivariate analysis, but not in multivariate regression analysis (32). The SPPB combines the measurement of these abilities and could therefore be useful to identify physical function parameters related to FOF. A recent study in healthy and mobility-limited community-dwelling older adults showed that the components of sarcopenia (i.e., low muscle mass, muscle strength and physical performance) independently contribute to increased FOF (23). Evidence on the relationship between muscle mass and FOF in neurological patients is lacking (17, 18, 31–35), even though neurological patients may be more likely to suffer from sarcopenia as it has recently been suggested that PD, motor neuron disease, Alzheimer's disease and sarcopenia may be part of an “extended neurodegenerative overlap syndrome” (42). Other factors associated with FOF may be the use of a walking aid (31, 43), whose incorrect use -which is frequent- may be associated with reduced stability (44), and psychological factors, such as depressive symptoms and anxiety (18, 43). This study aims to investigate the relationship between FOF and the SPPB, as well as components of sarcopenia (muscle mass and muscle strength) in a cohort of hospitalized neurogeriatric patients. This may help gain an improved understanding of FOF in this vulnerable patient cohort and propose aspects which may be studied further in longitudinal and intervention studies to evaluate factors that may be predictive of FOF or qualify as possible treatment targets.

## Materials and Methods

### Study Design

The data used for this study was obtained from the prospective, explorative observational multi-center study Cognitive and Motor Interaction in the Older Population (ComOn (45)). Briefly, the ComOn study investigated motor and cognitive deficits, as well as treatment outcomes in a large geriatric cohort. For this analysis, the cross-sectional data of the neurogeriatric patients from the baseline assessment in Kiel was used. The study was approved by the ethical committee of the Medical Faculty of Kiel University, and the study was conducted according to the principles of the Declaration of Helsinki.

### Patients

This study included 124 multimorbid inpatients that were recruited from September 2017 until March 2021 at their admission to the neurogeriatric ward of the University Hospital Schleswig-Holstein, Campus Kiel. The patients were geriatric, aged 60 years and older and suffered from at least two chronic conditions (45–47). Additional inclusion criteria were the ability to stand without personal aid for at least ten seconds and the capability to walk at least four meters (walking aids allowed) (45). Exclusion criteria were less than six points in the Montreal Cognitive Assessment (MoCA) (45, 48, 49), clinical diagnosis of severe deficits in consciousness, two or more falls during the previous week, history of or current drug abuse (except nicotine) and (corrected) visual acuity below 60% (as assessed with a Sloan Letter Chart for three-meter distance (45, 50)). All patients received their baseline assessment during the first two days of their hospital stay.

### Assessment of Body Composition

Bioelectrical impedance analysis (BIA, Akern Bia 101, SMT medical GmbH & Co. KG, Würzburg, Germany) was used to assess body composition. BIA measures the electric impedance (Z) and phase angle of an electric current going through the body, and the resistance (R) and reactance (Xc) can be calculated (51, 52). BIA was performed after a rest phase of at least ten minutes, positioning four electrodes (two each at the right arm and right leg) with the patient in a supine position (45, 52). In order to estimate the appendicular skeletal muscle mass (ASMM), we used the raw BIA measures and the cross-validated Sergi equation (36, 53):


$$\begin{array}{l} ASMM(kg)\;=\;-3,964\;+\;(0,227\times RI)\;+(0,095\times weight)\\ \;+\;(1,384\times sex)+(0,064\times \;Xc)\\ \;RI\;=\;\frac{height{\left(cm\right)}^{2}}{R(\Omega )},\;weight(kg),\\ sex:\;female\;=\;0,\;\;male=1 \end{array}$$

We adjusted the ASMM for body size by dividing by height squared (kg/m^2^) (36, 54, 55).

### Physical Performance and Grip Strength Assessment

To evaluate physical performance, the Short Physical Performance Battery (SPPB) was administered. The test includes the three balance tasks (each needed to be held for ten seconds) side-by-side stand (with both feet in a parallel position, semi-tandem stand with the front inner edge of one foot touching the rear inner edge of the other foot) and tandem stand (with one foot's tip toe touching the other foot's heel), two four-meter walks at a comfortable pace (the faster was used for analysis), and a five times sit-to-stand task (5xSST) from a chair that had to be performed as fast as possible without using the arms. Time was taken with a stopwatch and each test performance was ranked depending on successful execution and time needed to complete the tasks using a zero to four scale. The SPPB total score is the sum of the three subscores (walking duration, balance, 5xSST) and can range from zero to twelve with less than nine points being indicative for physical limitation. For the walking task, the use of a walking aid was recorded, balance tasks and 5xSST were performed without a walking aid. (19–21, 56).

To measure maximum grip strength, the Jamar hydraulic hand dynamometer (AFH, Lüdge, Germany) was used. The assessment was done for both hands according to the Southampton protocol (45, 57) with the adaptation that the arms were not rested on arm chairs. The highest score of the six taken grip strength measurements (each hand three times) was used for this analysis.

### Questionnaires/Screening Tests

The self-administered questionnaire Falls Efficacy Scale International (FES-I, (58)), was used to measure FOF. The patients rate their concern of falling in 16 specific activities of daily living (e.g., when reaching for something above the head or on the ground) on a scale from one (no concern) to four (maximum concern) and the total sum is then calculated.

Current depressive symptoms over the last two weeks were assessed using the Depression-im-Alter-Skala (DIA-S, (59) The questionnaire consists of ten items (e.g., “I am scared to say or do something wrong”), which the patients answer with yes or no. A maximum of ten points can be reached, a score of three points is considered marginal, scores of four or more points are considered suspicious of depressiveness.

The Mini Nutritional Assessment short-form (MNA-SF, (60, 61)) was used to assess nutritional status. It includes six items that each have two to four response categories ranked from zero to three. The items concern food intake, weight loss, mobility, psychological stress or acute disease, neuropsychological problems and Body Mass Index (BMI). A maximum of 14 points can be reached with eight to eleven points reflecting risk of malnutrition and less than or equal to seven points indicating malnourishment (61).

The MoCA (48) is a standardised neuropsychological screening test to assess cognitive function. A maximum of 30 points can be achieved, with <26 points suggesting a cognitive deficit (48).

Falls in the last three months were recorded in the interview as part of the geriatric screening according to Lachs et al. (62), and ≥1 fall (s) was considered a positive fall history. Age, gender, current medication and clinical diagnosis were derived from the medical records. Due to associations between falls and medication (e.g., antipsychotics, antidepressants, benzodiazepines) (63, 64) and potential associations between FOF and polypharmacy (43), number of medications was reported and included in the analyses.

### Statistical Analysis

For patients with one (*n* = 14, completeness of 94%) or two (*n* = 3, completeness of 88%) missing responses to single items of the FES-I we used individual mean imputation (65). We calculated the within subject average of completed items, imputed this average value for the missing item(s) and calculated the sum as an imputed FES-I total score for these patients. Patients were grouped into high and low FOF according to the recommended FES-I cut-off point, with a score of ≤ 22 reflecting low FOF and ≥23 high FOF (58). Group differences were examined using the Mann-Whitney *U*-test (as the assumption of normality for the t-test was rejected for all variables according to the results of the Shapiro-Wilk test) for continuous variables and Fisher's exact test for dichotomous variables. For the Mann-Whitney *U*-test the rank-biserial correlation was calculated as the effect size. Spearman's rho (ρ) was calculated to evaluate correlations between FES-I score and all variables. To address the main question of the relationship among FOF, SPPB and aspects of sarcopenia, three multiple linear regression models with FES-I score as dependent variable were computed using backward selection with AICc as selection criterion. In the first model the SPPB total score, ASMM/height^2^ (kg/m^2^) and grip strength (kg) were initially entered as predictors and age (years), gender, positive fall history, number of medications, use of a walking aid, DIA-S score and MoCA score were included as potential confounders. In the second model the SPPB subscores (walking duration, balance, 5xSST), ASMM/height^2^ (kg/m^2^) and grip strength (kg) were initially included as predictors and age (years), gender, positive fall history, number of medications, DIA-S score and MoCA score were included as potential confounders. In the third model the use of a walking aid was added to the second model as independent variable. All assumptions of multiple linear regression models were examined (linear relationship, no multicollinearity evaluated with variance inflation factors, independence of observations, no autocorrelation, homoscedasticity and multivariate normality). Results were considered significant if the two-sided p-value was ≤ 0.05. Data was analysed using JASP Version 0.16 statistical software (66) and R (R Foundation for Statistical Computing) Version 4.1.2 (67).

## Results

### Descriptive and Bivariate Statistics

A total of 124 patients were included in the study. Mean age was 77 years (± 6) and 66 patients (53%) were female. Most frequent primary diagnosis was PD, followed by atypical Parkinsonism, stroke, PNP and subcortical arteriosclerotic encephalopathy (SAE, Supplementary Table 1). Additional information on their multimorbidity profile and concomitant medication is provided in Supplementary Tables 2 and 3. 98 patients (79%) reported high FOF with a mean FES-I score of 36.5 (± 10.1), and 26 patients (21%) reported low FOF with a mean FES-I score of 19.2 (± 2.2).

Patients with high FOF had lower SPPB total scores ($\tilde{x}$ = 5.00, IQR= 3.00 vs. $\tilde{x}$ = 7.00, IQR = 2.00; rrb = −0.47, p < 0.001) and were more likely to use a walking aid (54.1 vs. 23.1%, p = 0.007). Details are provided in Table 1.

**Table 1:** Patients' characteristics and group comparisons.

	**Entire cohort *N* = 124**	**FES-I low (≤ 22 points)** ***N*** **= 26**			**FES-I high (≥ 23 points)** ***N*** **= 98**			***p*** **-values**		
		**Female *N* = 12**	**Male *N* = 14**	**Both genders *N* = 26**	**Female *N* = 54**	**Male *N* = 44**	**Both genders *N* = 98**			
	**x; IQR (x ± SD)**	**x; IQR (x ± SD)**	**x; IQR (x ± SD)**	**x; IQR (x ± SD)**	**x; IQR (x ± SD)**	**x; IQR (x ± SD)**	**x; IQR (x ± SD)**	**F ^a^**	**M ^b^**	**All ^c^**
Age (years)^°^	78.0; 7.0 (77.2 ± 6.0)	79.0; 2.5 (79.1 ± 2.6)	77.0; 5.5 (77.0 ± 6.4)	78.0; 4.5 (78.0 ± 5.0)	78.0; 8.8 (76.8 ± 6.2)	77.0; 5.0 (77.3 ± 6.2)	77.5; 7.0 (77.0 ± 6.2)	0.237	0.799	0.466
BMI (kg/m^2^)^°^	26.1; 5.8 (27.0 ± 5.3)	23.2; 5.4 (23.8 ± 3.6)	25.0; 3.9 (25.7 ± 3.4)	24.3; 4.9 (24.8 ± 3.6)	27.2; 5.0 (28.2 ± 6.1)	26.3; 6.7(26.8 ± 4.9)	26.8; 5.9 (27.5 ± 5.6)	0.009^*^	0.500	0.014^*^
MoCA (score)^°^	22.0; 6.0 (21.5 ± 4.0)	21.0; 6.3 (20.9 ± 5.1)	22.0; 6.5 (21.4 ± 4.3)	22.0; 6.8 (21.2 ± 4.6)	21.5; 5.0 (21.2 ± 3.8)	22.0; 5.3 (22.1 ± 3.9)	22.0; 5.8 (21.6 ± 3.9)	1.000	0.642	0.784
Number of diagnoses^°^	11.0; 7.0 (11.5 ± 5.3)	10.0; 5.3 (11.1 ± 6.2)	9.5; 5.8 (10.7 ± 4.9)	10.0; 5.8 (10.9 ± 5.4)	12.0; 6.5 (12.3 ± 5.3)	10.5; 8.0 (10.8 ± 5.4)	11.5; 7.0 (11.6 ± 5.4)	0.233	0.792	0.301
Number of medications^°^	7.5; 6.0 (7.3 ± 3.8)	5.5; 5.5 (6.2 ± 3.8)	6.5; 4.0 (6.4 ± 4.0)	6.0; 4.8 (6.3 ± 3.8)	8.0; 6.0 (8.0 ± 4.0)	7.0; 5.0 (6.9 ± 3.6)	8.0; 5.0 (7.5 ± 3.8)	0.179	0.535	0.154
ASMM/height (kg/m^2^)^°^	6.6; 1.6 (6.9 ± 1.2)	5.7; 1.1 (5.8 ± 0.6)	7.2; 1.2 (7.3 ± 0.8)	6.6; 1.5 (6.6 ± 1.1)	6.2; 0.9 (6.5 ± 1.0)	7.4; 1.6 (7.5 ± 1.3)	6.6; 1.8 (6.9 ± 1.3)	0.028^*^	0.660	0.359
Grip strength (kg)^°^	22.0; 14.0 (25.1 ± 10.7)	20.0; 6.3 (19.9 ± 7.2)	31.5; 17.5 (33.3 ± 10.9)	25.0; 11.8 (27.1 ± 11.4)	18.0; 5.8 (17.9 ± 5.4)	33.5; 13.1 (32.7 ± 9.5)	21.5; 14.0 (24.5 ± 10.5)	0.182	0.949	0.193
SPPB (score)^°^	6.0; 3.0 (5.7 ± 2.2)	7.0; 2.0 (7.0 ± 1.5)	7.5; 2.0 (7.1 ± 1.8)	7.0; 2.0 (7.0 ± 1.6)	5.0; 3.8 (5.0 ± 2.0)	6.0; 2.3 (5.7 ± 2.2)	5.0; 3.0 (5.3 ± 2.1)	0.003^*^	0.038^*^	<0.001^*^
FES-I (score)^°^	30.0; 19.3 (32.9 ± 11.5)	20.0; 2.8 (19.4 ± 2.3)	19.5; 3.8 (19.1 ± 2.1)	20.0; 3.8 (19.2 ± 2.2)	37.0; 15.9 (37.3 ± 9.3)	32.5; 15.5 (35.6 ± 11.1)	34.0; 16.9 (36.5 ± 10.1)	<0.001^*^	<0.001^*^	<0.001^*^
DIA-S (score)^°^	2.0; 4.0 (2.8 ± 2.5)	2.0; 2.8 (2.5 ± 2.2)	1.0; 2.0 (1.9 ± 2.8)	1.5; 3.0 (2.2 ± 2.5)	3.0; 4.0 (3.4 ± 2.7)	2.0; 2.0 (2.4 ± 2.2)	2.5; 4.0 (3.0 ± 2.5)	0.301	0.125	0.077
Positive fall history (%)**^+^**	61 (49.2)	5 (41.7)	6 (42.9)	11 (42.3)	22 (40.7)	28 (63.6)	50 (51.0)	1.000	0.218	0.510
Walking aid (%)**^+^**	59 (47.6)	4 (33.3)	2 (14.3)	6 (23.1)	30 (55.6)	23 (52.3)	53 (54.1)	0.210	0.015^*^	0.007^*^
MNA-SF (score)^°^	12.0; 3.0 (11.3 ± 2.3)	11.0; 3.5 (10.9 ± 2.0)	12.0; 4.0 (11.2 ± 2.7)	11.5; 4.0 (11.1 ± 2.4)	12.0; 2.0 (11.6 ± 2.2)	12.0; 3.8 (11.2 ± 2.5)	12.0; 3.0 (11.4 ± 2.3)	0.291	0.873	0.553

*Results are displayed as median; interquartile range (mean ± standard deviation) except for walking aid and positive fall history (displayed in total number and percentage). ASMM, appendicular skeletal muscle mass; BMI, body mass index; DIA-S, depression im alter-skala; FES-I, falls efficacy scale international; MNA-SF, mini nutritional assessment short-form; MoCA, montreal cognitive assessment; SPPB, short physical performance battery*.

° *Mann-Whitney-U Test;*

+ *Fisher's exact test*.

a *FES-I high compared to FES-i low (females only)*.

b *p < 0.05 FES-I high compared to FES-i low (males only)*.

c *FES-I high compared to FES-I low (entire cohort);*

* *p < 0.05*.

There was a significant inverse correlation between FES-I score and SPPB total score as well as all SPPB subscores. Significant correlations with FES-I total score were also found for DIA-S, and number of medications. Neither muscle mass nor grip strength correlated significantly with the FES-I score. Details are shown in Table 2.

**Table 2:** Spearman correlations with FES-I score.

	**Spearman**	
	**correlations with**	
	**FES-I score**	***p*** **-values**
ASMM/height (kg/m^2^)	−0.02	0.799
Grip strength (kg)	−0.12	0.195
SPPB total score	−0.37	<0.001^*^
SPPB walking duration subscore	−0.21	0.020^*^
SPPB 5xSST subscore	−0.27	0.003^*^
SPPB balance subscore	−0.35	<0.001^*^
Number of medications	0.25	0.006^*^
DIA-S score	0.29	0.001^*^

*ASMM, appendicular skeletal muscle mass; DIA-S, depression im alter-skala; FES-I, falls efficacy scale international; SPPB, short physical performance battery; 5xSST, five times sit-to-stand task,*

* *p < 0.05*.

### Regression Analyses

We first determined if SPPB total score was associated with the FES-I score after correction for cofactors. Variables entered as independent variables were: SPPB total score, ASMM/height^2^ (kg/m^2^), grip strength (kg), age (years), gender, positive fall history, number of medications, use of a walking aid, DIA-S score and MoCA score. In the first model using backward selection, the FES-I score was associated with SPPB total score (β = −0.22, *p* =0.015), use of a walking aid (β = 0.30, *p* = 0.001), number of medications (β = 0.16, *p* = 0.048) and age (β = −0.16, *p* = 0.047) and additionally included DIA-S score (β = 0.15, *p* = 0.066); adjusted R^2^ of the model was 0.27 (*p* < 0.001).

Next, we determined which SPPB subscores were associated with the FES-I score. Variables entered as independent variables were: SPPB subscores (walking duration, balance, 5xSST), ASMM/height, grip strength, age, gender, positive fall history, number of medications, DIA-S score and MoCA score. The FES-I score was associated with the SPPB balance (β = −0.29, *p* < 0.001) and SPPB 5xSST (β = −0.20, *p* = 0.015) subscores, DIA-S score (β = 0.17, *p* = 0.041), and number of medications (β = 0.18, *p* = 0.038) and adjusted R^2^ of the model was 0.22 (*p* < 0.001). When additionally correcting for the use of a walking aid, the explained variance increased (adjusted R^2^ = 0.29, *p* < 0.001) and the association between FES-I score and SPPB balance subscore became weaker (β = −0.16, *p* = 0.077) next to SPPB 5xSST (β = −0.17, *p* = 0.027) , DIA-S score (β = 0.16, *p* = 0.049), use of a walking aid (β = 0.30, *p* = 0.001), number of medications (β = 0.16, *p* = 0.046), and age (β = −0.14, *p* = 0.080). Details are shown in Tables 3 and 4.

**Table 3:** Multiple Regression Analyses including SPPB total score with FES-I score as dependent variable.

	**Model I**			
	**b (95% CI)**		**β**	**p**
SPPB total score	−1.17	(−2.12, −0.23)	−0.22	0.015^*^
DIA-S score	0.69	(−0.05, 1.42)	0.15	0.066
Walking aid	6.81	(2.73, 10.88)	0.30	0.001^*^
Number of medications	0.48	(0.00, 0.96)	0.16	0.048^*^
Age	−0.31	(−0.61, −0.00)	−0.16	0.047^*^

*ASMM, appendicular skeletal muscle mass; DIA-S, depression im alter-skala; FES-I, falls efficacy scale international; MoCA, montreal cognitive assessment; SPPB, short physical performance battery. Dichotome variables: gender (1 = female), walking aid (1 = yes), positive fall history (1 = yes). Variables entered as independent variables: SPPB total score, ASMM/height^2^ (kg/m^2^), grip strength (kg), age (years), gender, positive fall history, number of medications, use of a walking aid, DIA-S score and MoCA score. Model I Statistics: F (5, 118) = 10.23 (p < . 001), Adjusted R^2^ = 0.27*.

* *p < 0.05*.

**Table 4:** Multiple regression analyses including SPPB subscores with FES-I score as dependent variable (with and without including the use of a walking aid as independent variable).

	**Model II^+^**				**Model III^°^**			
	**b (95% CI)**		**β**	**p**	**b (95% CI)**		**β**	**p**
SPPB 5xSST subscore	−3.27	(−5.90, −0.64)	−0.20	0.015^*^	−2.90	(−5.41, −0.33)	−0.17	0.027^*^
SPPB balance subscore	−2.57	(−3.99, −1.15)	−0.29	<0.001^*^	−1.42	(−3.00, 0.16)	−0.16	0.077
Walking aid					6.85	(2.76, 10.94)	0.30	0.001^*^
DIA-S score	0.79	(0.03, 1.54)	0.17	0.041^*^	0.73	(0.003, 1.46)	0.16	0.049^*^
Number of medications	0.53	(0.03, 1.02)	0.18	0.038^*^	0.49	(0.01, 0.96)	0.16	0.046^*^
Age					−0.27	(−0.57, 0.03)	−0.14	0.080

*ASMM, appendicular skeletal muscle mass; DIA-S, depression im alter-skala; FES-I, falls efficacy scale international; MoCA, montreal cognitive assessment; SPPB, short physical performance battery; 5xSST, five times sit-to-stand task. Dichotome variables: gender (1 = female), walking aid (1 = yes), positive fall history (1 = yes)*.

+ *Variables entered as independent variables: SPPB 5xSST subscore, SPPB balance subscore, SPPB walking duration subscore, ASMM/height^2^ (kg/m^2^), grip strength (kg), age (years), gender, positive fall history, number of medications, DIA-S score and MoCA score*.

° *Variables entered as independent variables: SPPB 5xSST subscore, SPPB balance subscore, SPPB walking duration subscore, ASMM/height^2^ (kg/m^2^), grip strength (kg), age (years), gender, positive fall history, number of medications, use of a walking aid, DIA-S score and MoCA score. Model II Statistics: F (4, 119) = 9.81 (p < 0.001), Adjusted R^2^ = 0.22. Model III: F (6, 117) = 9.20 (p < 0.001), Adjusted R^2^ = 0.29*

* *p < 0.05*.

## Discussion

This study aimed to assess the relationship between FOF and aspects of physical performance and sarcopenia in a cohort of hospitalized neurogeriatric patients. Our main findings were that: (i) higher FOF was associated with decreased physical performance, especially with; (ii) decreased static balance; and (iii) decreased chair-rise performance. Moreover, number of medications, depressive symptoms and use of a walking aid were also associated with FOF, and the associations of FOF with static balance is weakened when considering use of a walking aid, although the physical task itself was performed without a walking aid.

Similar to studies in community-dwelling older adults (1, 22–24, 43), low physical performance was associated with higher FOF in our study cohort of hospitalized neurogeriatric patients. A study in patients with stroke found comparable results. The authors described an association between physical performance (also measured with the SPPB) and fall-related self-efficacy (26). A recent two-year longitudinal study in community-dwelling older adults demonstrated that by using two criteria: self-reported mobility disability and less than eight points in the SPPB total score, they were able to identify 82% of people at risk of developing FOF (25). Taken together, poor physical performance (measured with the SPPB) is strongly associated with, and may even be predictive for, FOF in different cohorts including neurogeriatric patients. If confirmed by subsequent studies, FOF should be taken into consideration for diagnostics and treatment routines in neurological patients with low SPPB total scores. As the SPPB is frequently used as part of the comprehensive geriatric assessment this may help identify patients with FOF in a clinical setting.

To understand which specific aspects of physical performance drive the relationship with FOF, walking duration, static balance and chair-rise performance were considered separately. As the use of a walking aid may have several underlying factors (balance confidence, balance ability, muscle strength, disease severity) which may influence the results, the analysis was performed twice, once with and once without including walking aid as independent variable. When the use of a walking aid was not considered, chair-rise performance and static balance ultimately showed a significant association with FOF. When including walking aid in the analysis the association between SPPB balance subscore and FOF was weakened. This may be because many patients with poor balance and FOF use a walking aid or because use of a walking aid may reflect low balance confidence and therefore may be associated with FOF. Our results support previous findings of an association between balance and FOF in patients with PD and stroke as well as in community-dwelling older adults (1, 32, 35). In contrast to other tools (e.g., the frequently used Berg Balance Scale (BBS) (32, 35, 68) that assesses balance more granularly) the SPPB balance tasks focus only on the assessment of static balance (dynamic balance aspects are measured merely indirectly within the walking and the 5xSST tasks). Although -again- no causality can be drawn from our results, our and previous studies' results suggest that improving balance may be an essential factor in the treatment portfolio against FOF also in this neurogeriatric cohort, as already shown for institutionalized older people with FOF (37).

The 5xSST can measure lower limb strength (36), and there are already studies in patients with stroke and PD available that demonstrate a relationship between the lower limb muscle strength and FOF (18, 34). Moreover, a study on patients with PD showed that self-reported difficulties in rising from a chair was significantly associated with FOF (17). Our analysis therefore confirms a widely recognized association in a patient cohort with overall advanced disease stages and existing multimorbidity, and also supports the hypothesis that in patients with FOF, particular attention must be paid to maintaining and improving muscle strength in the lower extremities in the context of self-directed exercise, allied health therapy and pharmacological management. Interestingly, muscle mass as assessed with BIA was not significantly associated with FOF in our analyses. This is in contrast to a study investigating the association between the individual components of sarcopenia and FOF in 26 healthy and 22 mobility-limited community-dwelling older adults that found low muscle mass to contribute to an increase of FOF (23). This discrepancy may be due to study population differences and different methods used to estimate muscle mass or FOF. Although these differences certainly need to be investigated in more detail, it may be inferred from the current results (and also from other studies and trials, e.g., (69)) that the focus in the treatment of FOF should be on strength training, and not (only) on muscle building *per se*, also in this cohort.

Also in contrast to a previous study in older adults (40), walking duration was not associated with FOF in multivariate analysis, but was only correlated in bivariate analysis. What is the most appropriate explanation for the lack of association between the SPPB walking duration subscore and FOF? Our patients were asked to walk at a comfortable pace, but it cannot be ruled out that some of them were extrinsically motivated by the test situation to walk faster than their usual pace. A previous study found that adults fearful of falling did walk slower than those who were not fearful, but similarly to the control group they were physically able to accelerate their gait speed when asked to do so (39). In line with this observation, studies in PD patients found an association between self-rated walking disability and FOF, but not between gait speed and FOF (32, 33). Additionally, a study in patients with PD found a significant difference in the time needed to perform the two turns during the Timed-up-go (TUG) including the stand-to-sit movement test between individuals with and without FOF, but not in the gait speed (38). Therefore, concerning FOF, this study also highlights the importance of the capability to perform transitions and does not see gait speed *per se* as relevantly predictive of this symptom. Another reason for the lack of significant association between SPPB walking duration subscore and FOF could be the simplicity of the walking condition (i.e., short distance of four meters, flat ward floor with no disturbances, presence of the examiner). A previous study found no significant difference in walking speed among individuals with and without FOF on flat ground. However, on an elevated walkway, walking speed of those with FOF decreased disproportionately, suggesting that walking speed of those individuals is only deteriorated under challenging conditions (41). In line with this idea, a systematic review pointed out the existing heterogeneity in gait speed measurement protocols (e.g., concerning the distance), which decreases comparability of studies. However, the four meter distance is the most frequently used distance to assess gait speed in older adults (10). Whatever the reasons are for this result, it seems likely overall that simple walking tasks can do little to define individuals with FOF.

The lack of association between grip strength and FOF that we observed in our analyses was already found in studies in older adults (70, 71). Therefore, it can be assumed with a high degree of confidence that measuring force at the upper extremities, which very likely contribute little to our mobility in space, may also not contribute relevantly to the detection and prediction of FOF.

Also interesting and in line with previous studies (32, 34), we did not find a significant association between previous falls and FOF, highlighting once again that FOF could also appear as a fall-independent condition.

Studies have already indicated that FOF is associated with walking aid use in community-dwelling older adults and adults with stroke (31, 43). In our analyses, the use of walking aids contributed the most to FOF of all determinants, even after including physical function parameters. There may be several reasons for this. For once, walking aid use may reflect poor balance confidence and/or low balance ability and may therefore be associated with FOF. Previous evidence has shown that use of a walking aid is an independent risk factor for falls (12) and a recent study revealed that walking aids are frequently used incorrectly, which was associated with reduced stability (44). The use of walking aids may be associated with FOF not only because they are used by patients with impaired physical function, but also because they reduce stability when used improperly, which may lead to an increase in FOF. Taken together, educating patients on the proper use of their walking aids and work with them to select the most suitable walking aid may have potential to reduce FOF. However, future studies are needed to further investigate the relationship between FOF and walking aid use.

Controversial evidence exists on the relationship between age and FOF. The studies that found an independent relationship described a positive association between age and FOF (2, 43). Contrarily, in our study cohort, younger age was associated with higher FOF in the first model and showed a trend toward significance in the third model. It may be hypothesized that younger patients on the neurogeriatric ward have a higher disease burden or have only recently received their diagnosis and haven't developed coping mechanisms yet. However, our findings differ in our models and are probably not representative of the general neurogeriatric cohort.

We also found a significant association between depressive symptoms and FOF (in all models, but the first one). A systematic review including studies on community-dwelling older adults found (although only weak) evidence on the relationship between depressive symptoms or depression and FOF (43), and a study in patients with stroke found an association between anxiety and FOF, but not depressive symptoms and FOF (18). A recent study in patients with PD showed that while depression predicted perceived consequences of falling, anxiety predicted fear-related activity avoidance (17). Depressive symptoms and FOF may be associated, because low self-efficacy is a symptom of depression and may be associated with low fall-efficacy, which is again related to FOF. Although studies' results vary, we assume that depressive symptoms and anxiety may play a role in the vicious cycle of FOF and activity avoidance and that treatment of FOF requires a holistic concept that must include also psychological components.

In our analysis, also the number of medications was associated with FOF. A higher number of medications may increase the risk of adverse drug events (e.g., hypotension, dizziness) (43, 63). The effect observed in our cohort may have been driven by specific drugs (e.g., antipsychotics, antidepressants, benzodiazepines) that have been linked to an increased risk of falling (63, 64). In our clinical practice we have indeed seen some patients who have reported a reduction in FOF after discontinuing antidepressants (e.g., citalopram). However, analyzing the relationship between specific medication and FOF is beyond the scope of this study and certainly needs larger cohort sizes, to be able to investigate the hen-egg problem as well.

Several limitations need to be addressed. First, due to the cross-sectional study design, we cannot draw conclusions regarding the causality of relationships. Second, this is a heterogeneous study cohort representing geriatric patients with predominating neurological disabilities with acute onsets and chronic disease courses (13), thus we cannot draw definite conclusions for patients with specific diseases and also cannot adjust the analyses for disease-related confounders (e.g., freezing in patients with PD). Third, we did not include measures of physical activity and anxiety, and we had to rely on self-reported retrospective falls assessment. Finally, possible influencing factors on the BIA measures (e.g., former dehydration, fluid and nutrition intake and body temperature) cannot be excluded with certainty. Although we have addressed these aspects in our assessment to the best of our ability, some variation in these parameters cannot be ruled out due to data collection in a clinical setting.

In conclusion, in geriatric patients with predominating neurological disabilities admitted to a University hospital, our preliminary results suggest that the SPPB, one of the most commonly used mobility assessment tasks in geriatric medicine, is associated with FOF. As a novel finding, the balance and 5xSST subscores of the SPPB contributed most to this effect. If confirmed by subsequent studies, it may thus be worthwhile to screen patients with low SPPB balance and 5xSST subscores for the presence of FOF and it may even be possible to predict the development of FOF. Although it is difficult to interpret the results from this cross-sectional study in terms of therapeutic effect, the following aspects may still be considered for future therapeutic studies and possibly also for clinical application: It may be beneficial to focus on (static) balance and muscle strength training especially of the lower limbs in neurogeriatric patients with FOF. Training patients to use their walking aids correctly, critical evaluation of medication especially in case of existing polypharmacy, and addressing mental aspects such as depressive symptoms might further help reduce FOF in this highly vulnerable cohort.

## Data Availability Statement

The raw data supporting the conclusions of this article will be made available by the authors, without undue reservation.

## Ethics Statement

The studies involving human participants were reviewed and approved by Ethics Commitee of the medical faculty of the Christian-Albrechts-University of Kiel. The patients/participants provided their written informed consent to participate in this study.

## Author Contributions

KS, JG, and WM were responsible for the conception and design of the study, as well as the analysis and interpretation of data. KS was responsible for drafting the manuscript. JG and WM made substantial contribution to the revision of the manuscript. TP and JW made substantial contributions to methodologies and revision of the manuscript. CM, JG, JW, and JK were responsible for the implementation of the database and organization of data and revised the manuscript critically. MR and KN were substantially involved in the acquisition of data and revised the manuscript critically. MD made substantial contributions to the revision of the manuscript. All authors contributed to the article and approved the submitted version.

## Funding

The study did not receive any external funding. We acknowledge financial support by DFG within the funding programme Open Access Publikationskosten.

## Conflict of Interest

The authors declare that the research was conducted in the absence of any commercial or financial relationships that could be construed as a potential conflict of interest.

## Publisher's Note

All claims expressed in this article are solely those of the authors and do not necessarily represent those of their affiliated organizations, or those of the publisher, the editors and the reviewers. Any product that may be evaluated in this article, or claim that may be made by its manufacturer, is not guaranteed or endorsed by the publisher.

## Abbreviations

ASMM: Appendicular skeletal muscle mass
BIA: Bioelectrical impedance analysis
ComOn: Cognitive and Motor Interaction in the Older Population
DIA-S: Depression Im Alter-Skala
FES-I: Falls Efficacy Scale International
FOF: Fear of falling
MNA-SF: Mini Nutritional Assessment short-form
MoCA: Montreal Cognitive Assessment
PD: Parkinson's Disease
PNP: Polyneuropathy
SAE: Subcortical arteriosclerotic encephalopathy
SPPB: Short Physical Performance Battery
5xSST: Five times sit-to-stand task.
