# Supplementary material for: Static Balance and Chair-Rise Performance in Neurogeriatric Patients: Promising Short Physical Performance Battery-Derived Predictors of Fear of Falling
Source: Front Med (Lausanne). 2022 Jun 21;9:904364. doi: 10.3389/fmed.2022.904364 (PMC9253572; doi:10.3389/fmed.2022.904364)
Supplement: Supplementary file 1 [file Data_Sheet_1.docx]

Supplementary Material

# Supplementary Tables (1-3)

| Diagnoses | N |
| --- | --- |
| Parkinson's disease | 48 |
| Atypical Parkinsonism | 21 |
| Stroke | 18 |
| Polyneuropathy | 10 |
| SAE including vascular dementia | 8 |
| Spinal stenosis | 4 |
| Functional movement disorder | 3 |
| Alzheimer´s disease | 2 |
| Motoneuron disease | 1 |
| Myopathy | 1 |
| Epilepsy | 1 |
| Amnestic MCI | 1 |
| Normal pressure hydrocephalus | 2 |
| Essential tremor | 1 |
| Myasthenia gravis | 1 |
| Cerebral venous sinus thrombosis | 1 |
| Drug-induced walking disorder | 1 |

**Supplementary Table 1. Main diagnoses of patients**

MCI, mild cognitive impairment;
SAE, subcortical arteriosclerotic encephalopathy.

**Supplementary Table 2. ICD-10-CM Codes of patients**

| ICD-10-CM Category | Designation | N patients (%) |
| --- | --- | --- |
| A00 - B99 | Certain infectious and parasitic diseases | 7 (5.6) |
| C00 - D49 | Neoplasms | 8 (6.5) |
| D50 - D89 | Diseases of the blood and blood-forming organs and certain disorders involving the immune mechanism | 8 (6.5) |
| E00 - E89 | Endocrine, nutritional and metabolic diseases | 81 (65.3) |
| F01 - F99 | Mental, Behavioral and Neurodevelopmental disorders | 51 (41.1) |
| G00 - G99 | Diseases of the nervous system | 114 (91.9) |
| H00 - H59 | Diseases of the eye and adnexa | 16 (12.9) |
| H60 - H95 | Diseases of the ear and mastoid process | 2 (1.6) |
| I00 - I99 | Diseases of the circulatory system | 91 (73.4) |
| J00 - J99 | Diseases of the respiratory system | 20 (16.1) |
| K00 - K95 | Diseases of the digestive system | 10 (8.1) |
| L00 - L99 | Diseases of the skin and subcutaneous tissue | 5 (4.0) |
| M00 - M99 | Diseases of the musculoskeletal system and connective tissue | 39 (31.5) |
| N00 - N99 | Diseases of the genitourinary system | 51 (41.1) |
| Q00 - Q99 | Congenital malformations, deformations and chromosomal abnormalities | 1 (0.8) |
| R00 - R99 | Symptoms, signs and abnormal clinical and laboratory findings, not elsewhere classified | 97 (78.2) |
| S00 - T88 | Injury, poisoning and certain other consequences of external causes | 16 (12.9) |
| U00 - U85 | Codes for special purposes | 119 (96.0) |
| Z00 - Z99 | Factors influencing health status and contact with health services | 94 (75.8) |

ICD-10-CM, International Classification of Diseases, Tenth Revision, Clinical Modification; N, number of patients
in total (and in %). The patients' exact ICD-10-CM Codes were grouped into the categories of diagnoses coded by
the first three characters of the ICD-10-CM Code. Note: Patients with more than one diagnosis in each ICD-10-CM Category appear only once in the corresponding patient count.

**Supplementary Table 3. Medication of patients (Anatomical Therapeutic Chemical Classification)**

| ATC-Code  (1^st^ and 2^nd^ level) | Designation | N Patients (%) |
| --- | --- | --- |
| A02 | Drugs for acid related disorders | 44 (35.5) |
| A03 | Drugs for functional gastrointestinal disorders | 3 (2.4) |
| A04 | Antiemetics and antinauseants | 1 (0.8) |
| A05 | Bile and liver therapy | 1 (0.8) |
| A06 | Drugs for constipation | 16 (12.9) |
| A07 | Antidiarrheals, intestinal antiinflammatory/antiinfective agents | 4 (3.2) |
| A09 | Digestives, incl. enzymes | 2 (1.6) |
| A10 | Drugs used in Diabetes | 15 (12.1) |
| A11 | Vitamins | 35 (28.2) |
| A12 | Mineral supplements | 13 (10.5) |
| B01 | Antithrombotic agents | 70 (56.5) |
| B03 | Antianemic preparations | 22 (17.7) |
| C01 | Cardiac therapy | 11 (8.9) |
| C02 | Antihypertensives | 1 (0.8) |
| C03 | Diuretics | 33 (26.6) |
| C07 | Beta blocking agents | 44 (35,5) |
| C08 | Calcium channel blockers | 25 (20.2) |
| C09 | Agents acting on the Renin-Angiotensin System | 60 (48.4%) |
| C10 | Lipid modifying agents | 41 (33.1) |
| G03 | Sex hormones and modulators of the genital system | 1 (0.8) |
| G04 | Urologicals | 31 (25) |
| H02 | Corticosteroids for systemic use | 4 (3.2) |
| H03 | Thyroid therapy | 25 (20.2) |
| J01 | Antibacterials for systemic use | 1 (0.8) |
| L02 | Endocrine therapy | 3 (2.4) |
| L04 | Immunosuppressants | 2 (1.6) |
| M01 | Antiinflammatory and antirheumatic products | 9 (7.3) |
| M03 | Muscle relaxants | 3 (2.4) |
| M04 | Antigout preparations | 7 (5.6) |
| M05 | Drugs for treatment of bone diseases | 7 (5.6) |
| M09 | Other drugs for disorders of the musculo-skeletal system | 1 (0.8) |
| N02 | Analgesics | 31 (25) |
| N03 | Antiepileptics | 20 (16.1) |
| N04 | Anti-parkinson drugs | 54 (43.5) |
| N05 | Psycholeptics | 15 (12.1) |
| N06 | Psychoanaleptics | 39 (31.5) |
| N07 | Other nervous system drugs | 6 (4.8) |
| R01 | Nasal preparations | 1 (0.8) |
| R03 | Drugs for obstructive airway diseases | 12 (9.7) |
| S01 | Ophtalmologicals | 9 (7.3) |
| V03 | All other therapeutic products | 2 (1.6) |
| V06 | General nutrients | 3 (2.4) |

ATC, Anatomical Therapeutic Chemical; N, number of patients in total (and in %). The first level of the ATC (first letter) refers to the anatomical region in which the drugs exert their effect and the subsequent two numbers (second level of the ATC classification) refer to the pharmacological/therapeutic subgroup. Note: Patients with more than one medication in each ATC-subgroup appear only once in the corresponding patient count.
